# Integrating and optimizing tonabersat in standard glioblastoma therapy: A preclinical study

**DOI:** 10.1371/journal.pone.0300552

**Published:** 2024-03-15

**Authors:** Velislava Zoteva, Valerie De Meulenaere, Christian Vanhove, Luc Leybaert, Robrecht Raedt, Leen Pieters, Anne Vral, Tom Boterberg, Karel Deblaere

**Affiliations:** 1 Department of Radiology, Ghent University, Ghent, Belgium; 2 IBiTech—Medisip—Infinity lab, Ghent University, Ghent, Belgium; 3 Physiology Group, Department of Basic and Applied Medical Sciences, Ghent University, Ghent, Belgium; 4 Department of Head and Skin, Ghent University, Ghent, Belgium; 5 Department of Human Structure and Repair, Ghent University, Ghent, Belgium; 6 Department of Radiation Oncology, Ghent University Hospital, Ghent, Belgium; Universidade Federal do ABC, BRAZIL

## Abstract

Glioblastoma (GB), a highly aggressive primary brain tumor, presents a poor prognosis despite the current standard therapy, including radiotherapy and temozolomide (TMZ) chemotherapy. Tumor microtubes involving connexin 43 (Cx43) contribute to glioma progression and therapy resistance, suggesting Cx43 inhibition as a potential treatment strategy. This research aims to explore the adjuvant potential of tonabersat, a Cx43 gap junction modulator and blood-brain barrier-penetrating compound, in combination with the standard of care for GB. In addition, different administration schedules and timings to optimize tonabersat’s therapeutic window are investigated. The F98 Fischer rat model will be utilized to investigate tonabersat’s impact in a clinically relevant setting, by incorporating fractionated radiotherapy (three fractions of 9 Gy) and TMZ chemotherapy (29 mg/kg). This study will evaluate tonabersat’s impact on tumor growth, survival, and treatment response through advanced imaging (CE T1-w MRI) and histological analysis. Results show extended survival in rats receiving tonabersat with standard care, highlighting its adjuvant potential. Daily tonabersat administration, both preceding and following radiotherapy, emerges as a promising approach for maximizing survival outcomes. The study suggests tonabersat’s potential to reduce tumor invasiveness, providing a new avenue for GB treatment. In conclusion, this preclinical investigation highlights tonabersat’s potential as an effective adjuvant treatment for GB, and its established safety profile from clinical trials in migraine treatment presents a promising foundation for further exploration.

## Introduction

Glioblastoma (GB) is a highly aggressive primary brain tumor in adults, characterized by an exceedingly poor prognosis. The typical median survival post-diagnosis is only 12–15 months, with a 5-year survival rate of 5% [1]. Approximately 80% of all malignant primary brain tumors, including GB, originate from glial stem or progenitor cells and are collectively referred to as glioma [2]. Glial cells, including microglia, astrocytes, and oligodendrocytes, are the most abundant cells in the central nervous system. Their main functions include supporting neurotransmission, maintaining central nervous system homeostasis, and assisting in brain development [3, 4]. Consequently, the dysregulation or dysfunction of glial cells, particularly astrocytes, can contribute to the pathogenesis of various neurological conditions, spanning from multiple sclerosis to Parkinson’s disease and gliomas [5]. GB represents more than half of all gliomas and is classified as a grade 4 tumor according to the 2021 World Health Organization (WHO) classification (i.e., grades 1 to 4 from least to most aggressive behavior), due to its highly infiltrative and lethal nature [1, 6]. Magnetic resonance imaging (MRI) with gadolinium (Gd)-based contrast is the primary diagnostic tool for GB, followed by histological and molecular analysis for confirmation [1, 7]. GB can develop rapidly *de novo*, termed primary GB, or evolve from low-grade astrocytoma, termed secondary GB. Primary GB, accounting for approximately 90% of cases, typically occurs in elderly patients, exhibits a high degree of necrosis, and has a poor prognosis [8]. In contrast, secondary GB is more prevalent in younger patients and is associated with a better prognosis and lower necrosis levels. Differentiating between both is only possible through genetic and epigenetic profile analyses rather than histological examination [8]. Since 2005, the standard therapy for GB, well-known as the Stupp protocol, includes surgical resection, fractionated radiotherapy (30 fractions of 2 Gy) and concomitant and adjuvant temozolomide (TMZ) chemotherapy [9]. Despite this multidisciplinary approach, the persistently poor prognoses observed in patients underscore the necessity for novel therapeutic procedures. The primary challenges contributing to minimal treatment efficacy are high recurrence rates due to intra-tumoral heterogeneity, therapy resistance, and incomplete surgical resection caused by GB’s invasive nature in brain tissue [10].

Recent studies have discovered significant roles played by gap junctions, which consist of transmembrane connexin (Cx) proteins, within the context of glioma [11–13]. Cxs are protein subunits that oligomerize into hexameric structures, known as hemichannels. The fusion of two hemichannels, located within the membranes of neighboring cells, culminates in the formation of gap junctional channels [13]. These gap junctional channels facilitate direct gap junctional intercellular communication (GJIC), permitting the transfer of small molecules, second messengers, ions, microRNAs, and electrical signals. Long microtube-associated gap junctions composed of connexin 43 (Cx43), the most ubiquitous connexin protein, have been observed in GB cells and are referred to as tumor microtubes [11]. GB cells can interconnect via these tumor microtubes to establish a malignant multicellular network that facilitates brain invasion, proliferation and enables direct GJIC [11, 14, 15]. Studies illustrated that this Cx43-mediated GJIC manifests both between glioma cells and between reactive astrocytes, as well as in a heterocellular manner between glioma cells and reactive astrocytes [12, 16, 17]. While GJIC is essential under physiological conditions for maintaining tissue homeostasis, regulating cell growth, and coordinating cellular functions, in the context of glioma, it contributes to the formation of passages for brain microinvasion and therapy resistance, ultimately promoting GB survival [15–19]. This therapy resistance involves the sequestration of potentially toxic levels of calcium and other metabolites, which would otherwise reach lethal concentrations due to radio- or chemotherapy [20–23]. Hence, the inhibition of GJIC between tumor cells and cells of their microenvironment could be promising in decreasing glioma cell proliferation, invasion, and resistance to therapy.

In pursuit of enhancing overall patient survival and improving the quality of life for individuals with GB, we will investigate a novel adjuvant therapeutic, a Cx43 gap junction modulator called tonabersat, in preclinical phase to complement the standard of care. Tonabersat is a benzopyran compound that effectively passes the blood-brain barrier. It exhibits selective and specific binding to a unique stereoselective site in astrocytes, leading to the inhibition of hemichannel- (i.e., a single pair of a gap junction) or gap junction-mediated processes, dependent on the administration dose [24–26]. Research has shown that, under pathological conditions, tonabersat effectively inhibits Cx43 hemichannel opening and reduces Cx43 gap junction coupling in *in vitro* studies [27]. Moreover, preclinical investigations and clinical trials have demonstrated that tonabersat blocks cortical spreading depression, a mechanism associated with depolarizing brain waves during migraine aura. Furthermore, tonabersat has progressed to phase II clinical trials as a promising treatment for migraine, with no reported severe adverse events and excellent patient tolerability [27–29]. In addition, preclinical results have highlighted tonabersat’s anticonvulsant properties, leading to its selection as a candidate for phase I clinical studies aimed at treating epilepsy [30, 31]. Moreover, tonabersat’s potential is not limited to its direct effect on Cxs. Preclinical studies have also indicated its capability to interact with conventional therapies, enhancing their cytotoxic effects and circumventing treatment resistance mechanisms [26, 32]. This raises the possibility of tonabersat serving as a potent adjuvant to the standard of care for GB, potentially leading to improved treatment outcomes.

The F98 Fischer rat model is one of the most widely used animal models for conducting GB studies. This model is weakly immunogenic and gives rise to heterogeneous infiltrative tumors that closely mimic the malignant and morphological characteristics observed in human GB [33–36]. Prior investigations in our research group yielded promising results concerning the therapeutic potential of tonabersat in the context of GB treatment using the F98 rat model [32]. This study demonstrated that combining tonabersat with radiotherapy (a single dose of 20 Gy) and TMZ chemotherapy (29 mg/kg) led to significant lower GB volumes when compared to the group treated solely with standard therapy. Additionally, monotherapy with tonabersat did not yield favorable outcomes, highlighting the importance of employing combination therapeutic strategies to maximize GB treatment efficacy [32, 37, 38].

In this study, we will use the newly optimized and standardized F98 GB rat model, designed to incorporate the standard of care protocol. This model integrates MRI-guided fractionated radiotherapy (three fractions of 9 Gy) and concomitant TMZ chemotherapy, replicating the clinical situation more closely [39]. This approach represents an enhanced methodology compared to the single-dose irradiation previously employed by De Meulenaere et al. [32]. The primary aim is to investigate the adjuvant effect of tonabersat when combined with the standard of care. Notably, the three 9 Gy fractions applied in the animal model represent a biologically equivalent dose to the 30 fractions of 2 Gy conventionally applied in human GB treatment [9]. Subsequently, our study will delve into assessing tonabersat’s sensitizing effects to chemo- or radiotherapy, by deconstructing the standard care into its components while introducing tonabersat into each treatment group. In the second phase of the study, we will examine the impact of different tonabersat administration schedules. Specifically, comparing a continuous administration, dosing on a 7/7 days per week basis, to a recovery period approach, administering tonabersat on a 5/7 days per week regimen (i.e, with a 2-day drug-free recovery period), to assess their respective effects on treatment outcomes. Additionally, by investigating the timing of administration and assessing its early or late effect in the treatment schedule (i.e., tonabersat administration concurrent with radiotherapy vs post-radiotherapy tonabersat administration), we are attempting to unravel the optimal therapeutic window for tonabersat intervention.

## Materials & methods

### Cell culture

The F98 cell line, obtained from the American Type Culture Collection (CRL-2397™, Virginia, United States), was established through the administration of a single dose of N-ethyl-N-nitrosourea (ENU) into pregnant Fischer rats during the 20th day of gestation. Subsequently, the offspring developed a brain tumor that was harvested and maintained in culture [40]. All chemical products were purchased from Thermo Fisher Scientific (Massachusetts, United States) unless specified otherwise. The F98 cancer cells were cultured in monolayers using Dulbecco’s modified Eagle’s medium (DMEM), supplemented with 10% Fetal Bovine Serum (FBS), 1% penicillin/streptomycin antibiotics, 0.1% Amphotericin B and 1% sodium pyruvate (100 mM, Sigma Aldrich, Missouri, United States), and maintained at 37˚C and 5% CO_2_.

### Ethics statement and animal maintenance

This research protocol received approval from the Ghent University ethics committee for animal experiments (ECD 21/06). The care and handling of all rats adhered to European guidelines (Directive 2010/63/EU) and involved housing them under controlled environmental conditions, including a standard 12-hour light/dark cycle, temperature maintenance within the range of 20˚C to 24˚C, and relative humidity maintained between 40% and 70%. Daily monitoring was conducted, and the rats were provided with food and water ad libitum.

### Experimental setup: The F98 GB rat model

#### Intracerebral implantation

Female Fischer rats (n = 44) (F344/IsoCrl, Charles River®) aged 10 ± 0.13 weeks (mean ± SD) with a mean body weight of 149 ± 6g were anesthetized with isoflurane (induction 5%, maintenance 2%) mixed with oxygen (0,5L/min), and immobilized using a stereotaxic frame (Model 902 Dual Small Animal Stereotaxic frame, Kopf1). The rectal temperature was maintained at 37.0±0.5˚C via a feedback-controlled heating pad connected to a rectal probe. Subsequently, the rat’s head was shaved, and a scalp incision was made to expose the skull. A 1 mm hole was drilled (diamond drill, Dremel®) in the skull, 8 mm posterior and 4.5 mm lateral to the bregma. A stereotactically guided syringe (29G × 12.7 mm) containing a 5 μL PBS cell suspension with 5000 F98 glioma cells was slowly injected at a depth of 4.1 mm (right entorhinal cortex) at a rate of 0.5 μL/min using a micro syringe pump controller (Micro 4TM, World Precision Instruments, Sarasota, USA). Post-injection, the syringe remained in place for 5 minutes before being slowly withdrawn in six steps, each involving a 0.7 mm retraction and a 2-minute pause, totalling 12 minutes. The burr hole was sealed with simplex rapid powder (Kemdent®, Swindon, England), the incision was sutured, and a subcutaneous injection of meloxicam (1 mL/kg) was administered. Additionally, subcutaneous injections of sodium Chloride 0.9% w/v and Glucose 5% w/v solution (2mL/kg) were given to prevent dehydration. Finally, Neobacitracine (BePharBel Manufacturing, Courcelles, Belgium) was applied locally as an antibiotic.

#### Confirmation of GB growth with MRI

Ten days post-intracerebral implantation, MRI scans were conducted for tumor validation within the brain, utilizing a 7T μMRI system (PharmaScan 70/16, Bruker BioSpin, Ettlingen, Germany). Hence, rats were anesthetized with isoflurane (induction 5%, maintenance 2%) mixed with oxygen (0.5L/min). Subsequently, an intravenous injection (IV) of a Gd-based contrast agent (Gadovist®, Bayer, Germany; 1mmol/kg) was administered via a tail vein. Then, the anesthetized rat was securely positioned on a heating pad in the restrained bed, while the body temperature was maintained at 37°C. A rat brain surface coil (Rapid Biomedical, Rimpar, Germany) was secured around the head and the bed was positioned in a 72 mm rat whole-body transmitter coil (Rapid Biomedical, Rimpar, Germany), and a contrast-enhanced (CE) T1-weighted (T1-w) spin echo sequence (117 μm in-plane resolution, TR/TE 1539/9.7 ms, 3 averages, TA 4’15”) was acquired. Confirmation of GB growth was established when the tumor diameter measured 2.5–3.5mm on the CE T1-w MR image. This day was designated as day 0, at which point each animal was randomly assigned to a treatment group.

#### Treatment groups

Seven distinct animal groups were established for this study.

*Control group*: *Standard GB therapy*. The rats in the control group (n = 7) received standard GB therapy, which includes fractionated radiotherapy (3 fractions of 9Gy) and TMZ chemotherapy (29mg/kg).

*Tonabersat + standard of care group*. The rats in this group (n = 7) received standard GB therapy (i.e., fractionated radiotherapy and TMZ chemotherapy) in combination with daily tonabersat injections (10mg/kg) on a 7/7 days/week schedule starting on the day of GB confirmation (i.e., day 0). In the second phase of the study, wherein diverse administration schedules of tonabersat are explored, the data from this group was reused, aligning with the 3Rs principle (Replacement, Reduction, and Refinement, which provide ethical guidelines for the responsible use of animals in scientific research). To ensure clarity, the group was renamed as ’tonabersat 7/7’ in the second phase of the study.

*Tonabersat + TMZ group*. The rats in this group (n = 6) received TMZ chemotherapy together with daily tonabersat injections, starting on the day of GB confirmation (i.e., day 0). This group was included to assess tonabersat’s possible sensitizing effects to chemotherapy.

*Tonabersat + radiotherapy group*. The rats in this group (n = 7) received fractionated radiotherapy together with daily tonabersat injections, starting on the day of GB confirmation (i.e., day 0). This groups was included to assess tonabersat’s possible sensitizing effects to radiotherapy.

*Tonabersat 5/7 group*. The rats in this group (n = 5) received standard GB therapy (i.e., fractionated radiotherapy and TMZ chemotherapy) in combination with tonabersat injections administered solely on weekdays (i.e., a 5/7 days per week regimen), incorporating a 2-day drug-free recovery time interval. The tonabersat injections started on the day of GB confirmation (i.e., day 0).

*Tonabersat during radiotherapy group*. The rats in this group (n = 6) received standard GB therapy (i.e., fractionated radiotherapy and TMZ chemotherapy) in combination with tonabersat administration concurrent with the radiotherapy protocol, starting on the day of GB confirmation (i.e., from day 0–5). This group was included to investigate the timing of tonabersat administration and to assess its possible early therapeutic effect in the treatment schedule.

*Tonabersat after radiotherapy group*. The rats in this group (n = 6) received standard GB therapy (i.e., fractionated radiotherapy and TMZ chemotherapy) in combination with daily post-radiotherapy tonabersat injections (i.e., starting from day 6 onwards post-GB confirmation). This group was included to investigate the timing of tonabersat administration and to assess its possible late therapeutic effect in the treatment schedule.

#### Treatment protocol

*Fractionated MRI-based radiotherapy (3 x 9 Gy)*. The rats receiving fractionated radiotherapy, initiated their treatment on the day following tumor confirmation (i.e., day 1). The MRI-guided radiotherapy treatment was performed using the SARRP (XStrahl, United Kingdom). Initially, the rats were anesthetized with isoflurane (induction 5%, maintenance 2%) mixed with oxygen (0.5L/min), and an IV injection of Gadovist® was administered via a tail vein. Subsequently, the rats were immobilized on a multimodality bed for a CE T1-w MRI scan. The rats were then transferred to the four-axis robotic positioning table of the SARRP. A treatment planning CT was acquired, encompassing a total of 720 projections taken over 360° (voltage X-ray source: 60 kV, tube current: 600 μA, and aluminium filter: 1 mm). Subsequently, the CT data were reconstructed with an isotropic voxel size of 0.2 mm and imported into the treatment planning software Muriplan (Version 3.0.0, Xstrahl, UK), for manual segmentation to distinguish air, soft tissue, and bone. The obtained MR image was precisely co-registered with the planning CT, with the center of the contrast-enhanced tumor region on the CE T1-w MR image serving as the irradiation target (i.e., isocenter). Using a 5 x 5 mm collimator, a total dose of 27 Gy was administered in 3 fractions of 9 Gy each (voltage X-ray source: 220kV; tube current: 13 mA; copper filter of 0.15 mm) on day 1, day 3, and day 5, by applying a single beam parallel to and in line with the inoculation route, in addition to two non-coplanar arc beams (arc rotations of 120°).

*TMZ chemotherapy*. The rats receiving TMZ chemotherapy, initiated their treatment on the day after tumor confirmation (i.e., day 1), which, in the majority of groups, coincided with the initiation of radiotherapy. Each rat received IP injections of TMZ (29mg/kg; MedChem Express, New Jersey, United States), dissolved in 20% DMSO (Sigma Aldrich, Missouri, United States) and diluted with saline to 1mL for five consecutive days (i.e., day 1–5). Before the injection, the rat was anesthetized with isoflurane (induction 5%, maintenance 2%) mixed with oxygen (0.5L/min).

*Tonabersat therapy*. The rats undergoing tonabersat therapy received IP injections of tonabersat (SB-220453) (10mg/kg; MedChem Express, New Jersey, United States), solubilized in a solution containing 10% DMSO (Sigma Aldrich, Missouri, United States) and 10% tween20 (Sigma Aldrich, Missouri, United States), which were then diluted with PBS to a final volume of 1mL. Tonabersat injections were administered in accordance with the specified schedules outlined in the respective treatment groups. These injections were administered until humane endpoints were reached, except for the rats receiving tonabersat exclusively during radiotherapy (administration ceased from day 6 onwards). Before the injection, the rats were anesthetized with isoflurane (induction 5%, maintenance 2%) mixed with oxygen (0.5L/min).

#### MRI follow-up & euthanasia (humane endpoints)

To evaluate treatment response, CE T1-w MRI sequences were performed every three days until humane endpoints were reached (i.e., on day 3, day 6, day 9, …). During the experimental setup, all rats were daily examined for specific clinical and behavioral signs of distress, including balance problems, diminished activity, lack of grooming, weight loss (a reduction of more than 20% of their maximum weight), and/or a hunchback posture. Additionally, the CE T1-w MRI scans were used to monitor GB growth (euthanasia when tumor volume was >40% of total brain volume) and pathological changes, including the invasion of a complete cerebral hemisphere, extreme growth of an extra-axial or extracranial tumor (> 10% of total tumor volume), midline shift, or expansion of the ventricles. When humane endpoints were reached, instantaneous euthanasia was performed by an IV injection of natrium pentobarbital (100 mg/kg; Euthanimal 20% (200 mg/mL); Hoogstraten, Belgium). An overview of the humane endpoints for each rat can be found in the supplementary data (S1 Table).

### Histological analysis

Following euthanasia, the brain of one rat from each experimental group was isolated. The isolated brain was immersed in 4% paraformaldehyde for 24 hours and subsequently embedded in paraffin. For the histological verification of GB development, the cerebrum was sectioned in 5μm slices and stained with hematoxylin and eosin (H&E). The presence of GB tumor in the right entorhinal cortex, the pattern of infiltration, and intrinsic tumor characteristics were investigated.

Moreover, immunohistochemical staining for Cx43 and glial fibrillary acidic protein (GFAP) was performed on formalin-fixed, paraffin-embedded slices to evaluate Cx43 expression and reactive astrocytes, respectively. Sections were subjected to a 30 min incubation with 1% Bovine serum albumin (BSA), mixed with either 5% normal swine serum (for Cx43) or 5% normal rabbit serum (for GFAP), followed by incubation with the primary antibodies: rabbit monoclonal (Cx43: 1/2000, 2h, Abcam ab11370) or mouse monoclonal (GFAP: 1/400, overnight, Thermo- Fisher, MA5-12023). Subsequently, sections were incubated with biotinylated secondary antibodies (1/200, 30 min), followed by a streptavidin-peroxidase complex incubation (1/200, 30 min), and 3,30- diaminobenzidine (DAB) peroxidase solution (10 min). Finally, the sections were counterstained with hematoxylin and coverslipped using mounting medium (4111, Richard-Allan Scientific, Thermo Fisher Scientific). High-resolution digital scans of all sections (40× magnification) were acquired using a virtual scanning microscope (Olympus BX51, Olympus Belgium SA/NV, Berchem, Belgium).

### Statistical analysis

Sample size calculations were performed using the resource equation approach, which calculates the minimum and maximum number of required animals. This approach is suitable when assuming the standard deviation, the effect size, or when analyzing multiple endpoints becomes challenging or involves complex statistical procedures [41, 42].

Tumor volumes were quantified from the CE T1-w MR scans using the Fiji software for each timepoint. This entailed manual delineation of contrast-enhancing regions on each image slice, with subsequent multiplication of these areas by the slice thickness (0.6 mm). To analyze potential group differences in tumor volumes on day 0, we employed the Kruskal-Wallis test. It is crucial to ensure that the initial tumor volumes of the animals are comparable, so variations between the animals are minimized and meaningful comparisons in the study are enabled.

Estimated mean tumor volumes were obtained through a linear mixed model, adjusted for the baseline tumor volume of each animal (volume at day 0). Due to variable euthanasia timepoints, the dataset exhibited missing data by the missing-at-random principle. The model incorporated the natural logarithm of tumor volume as the response variable. In the model’s fixed component, the treatment groups, the time elapsed since tumor confirmation, their interaction, and the natural logarithm of the baseline tumor volume were included. The random intercept included the individual animals, considering the repeated measurements within the same animal. Results were graphically depicted, showing estimated mean tumor volumes (in mm^3^) at the different measured timepoints (in days) for each treatment group. Two estimated mean tumor volume increases were calculated per treatment group (from day 0 until day 15, and from day 15 onwards), accompanied by their corresponding 95% confidence intervals. In addition, a linear mixed model analysis was employed to compare body weights across the different timepoints within the various treatment groups.

Furthermore, survival analyses were conducted utilizing the Kaplan-Meijer method, followed by a Holm post-hoc analysis. Survival time (in days) was defined as the duration between tumor confirmation and the occurrence of humane endpoints, necessitating euthanasia. Statistical significance was set at p < 0.05, and all statistical analyses were performed using RStudio version 3.5.2 (source code available at https://github.com/VelislavaZoteva/PlosOne_Tonabersat.git).

## Results

### Evaluation of treatment response based on tumor volumes

#### Estimated tumor volume over time

All animals developed a GB at the site of inoculation, with an average timespan from the time of inoculation until GB confirmation of 10±2 days. The Kruskal-Wallis test showed no statistically significant differences in tumor volume on the day of GB confirmation (i.e., day 0) across the seven experimental groups (p-value = 0.70). In the six groups subjected to fractionated radiotherapy, a period of tumor growth stabilization was observed, followed by an exponential increase (supplementary data, S1 and S2 Figs). This stabilization state persisted from day 3 to day 15 (mean R^2^-value = 0.80). Subsequently, the GB’s exponential growth commenced on day 18 onwards (mean R^2^-value = 0.96). Conversely, the group receiving the combination of tonabersat and TMZ displayed no tumor stabilization state but rather exhibited an exponential increase from the onset (i.e., day 0, R^2^-value = 0.94).

**Fig 1:**
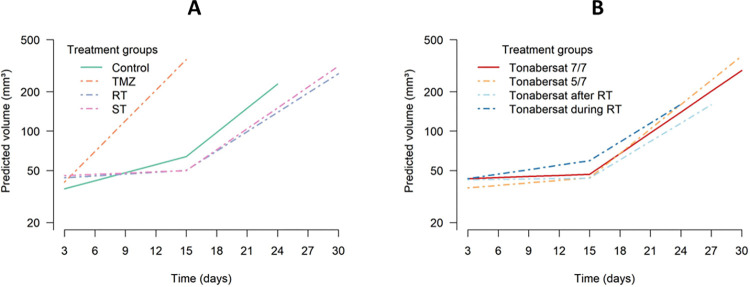
Estimated mean GB volumes as a function of time from inoculation until humane endpoints are reached. Tumor volume estimates were obtained through a linear mixed model, adjusted for the baseline tumor volume. (A) Visualization of the rat groups assessing the therapeutic added value of tonabersat in combination with TMZ and/or RT. Mean GB volume increases were estimated from day 0–15 and from day 15 onwards, respectively: ‘control’ group (4.86%, 15.24%), ‘tonabersat + TMZ’ group (orange dashed line, 19.67%, human endpoint reached), ‘tonabersat + RT’ group (purple dashed line, 1.08%, 11.99%), ‘tonabersat + ST’ group (pink dashed line, 0.74%, 13.05%). Significant differences in estimated mean tumor volume increases were only observed until day 15 between the group ‘tonabersat + TMZ’ and the groups ‘control’ (p-value < 0.0001), ‘tonabersat + radiotherapy’ (p-value < 0.0001), and ‘tonabersat + standard of care’ (p-value < 0.0001). (B) Visualization of the rat groups investigating the impact of tonabersat on various treatment schedules and timings during GB therapy. Mean GB volume increases were estimated from day 0–15 and from day 15 onwards, respectively: ‘tonabersat 5/7’ group (1.50%, 15.36%), ‘tonabersat during radiotherapy’ group (0.22%, 11.39%), and ‘tonabersat after radiotherapy’ group ‘2.65%, 11.67%). No significant differences in mean tumor volume increases were observed between these groups, except borderline significant results were observed from day 15 onwards between the ‘tonabersat 5/7’ group and ‘tonabersat during radiotherapy’ group (p-value = 0.07). Results show estimated mean tumor volumes (in mm^3^) at the different measured timepoints (in days) for each treatment group. Abbreviations: standard of care (ST), temozolomide (TMZ), radiotherapy (RT).

Fig 1 illustrates the estimated mean tumor volumes as a function of time (i.e., days from tumor confirmation until humane endpoints were reached) for the groups assessing tonabersat’s therapeutic effects in combination with RT, TMZ and standard of care (Fig 1A), and for the groups examining the impact of different tonabersat treatment schedules (Fig 1B).

The estimated mean tumor volume increase from day 0 to day 15 were as follows: for the control group 4.86% (95% CI: 2.88–6.84), for the ‘tonabersat + TMZ’ group 19.67% (95% CI: 16.34–22.94), for the ‘tonabersat + radiotherapy’ group 1.08% (95% CI: 0–2.88), for the ‘tonabersat + standard of care’ group 0.74% (95% CI: 0–2.51), for the ‘tonabersat 5/7’ group 1.5% (95% CI: 0–3.36), for the ‘tonabersat after radiotherapy’ group 0.22% (95% CI: 0–1.93), and for the ‘tonabersat during radiotherapy’ group 2.65% (95% CI: 0.86–4.46).

In addition, the estimated mean tumor volume increase from day 15 onwards were as follows: for the control group 15.24% (95% CI: 11.73–18.69), for the ‘tonabersat + radiotherapy’ group 11.99% (95% CI: 9.82–14.15), for the ‘tonabersat + standard of care’ group 13.05% (95% CI: 11.10–15.00), for the ‘tonabersat 5/7’group 15.36% (95% CI: 13.29–17.45), for the ‘tonabersat after radiotherapy’ group 11.39% (95% CI: 9.08–13.60), and for the ‘tonabersat during radiotherapy’ group 11.67% (95% CI: 8.36–14.84).

Significant higher mean tumor volume increases were observed until day 15 in the ‘tonabersat + TMZ’ group compared to the groups ‘control’ (p-value < 0.0001), ‘tonabersat + radiotherapy’ (p-value < 0.0001), and ‘tonabersat + standard of care’ (p-value < 0.0001). No significant differences in estimated mean tumor volume increases were observed from day 15 onwards between the control group, the ‘tonabersat + standard of care’ group, and the ‘tonabersat + radiotherapy’ group.

Moreover, no significant differences in estimated mean tumor volume increases were observed until day 15 between the ‘tonabersat 7/7’, the ‘tonabersat 5/7’, and ‘tonabersat after radiotherapy’. Also from day 15 onwards, no significant differences were found between these four groups. However, borderline significant results were observed when comparing the ‘tonabersat 5/7’ with the ‘tonabersat during radiotherapy’ group (p-value = 0.07).

#### Visual evaluation of tumor growth over time

**Fig 2:**
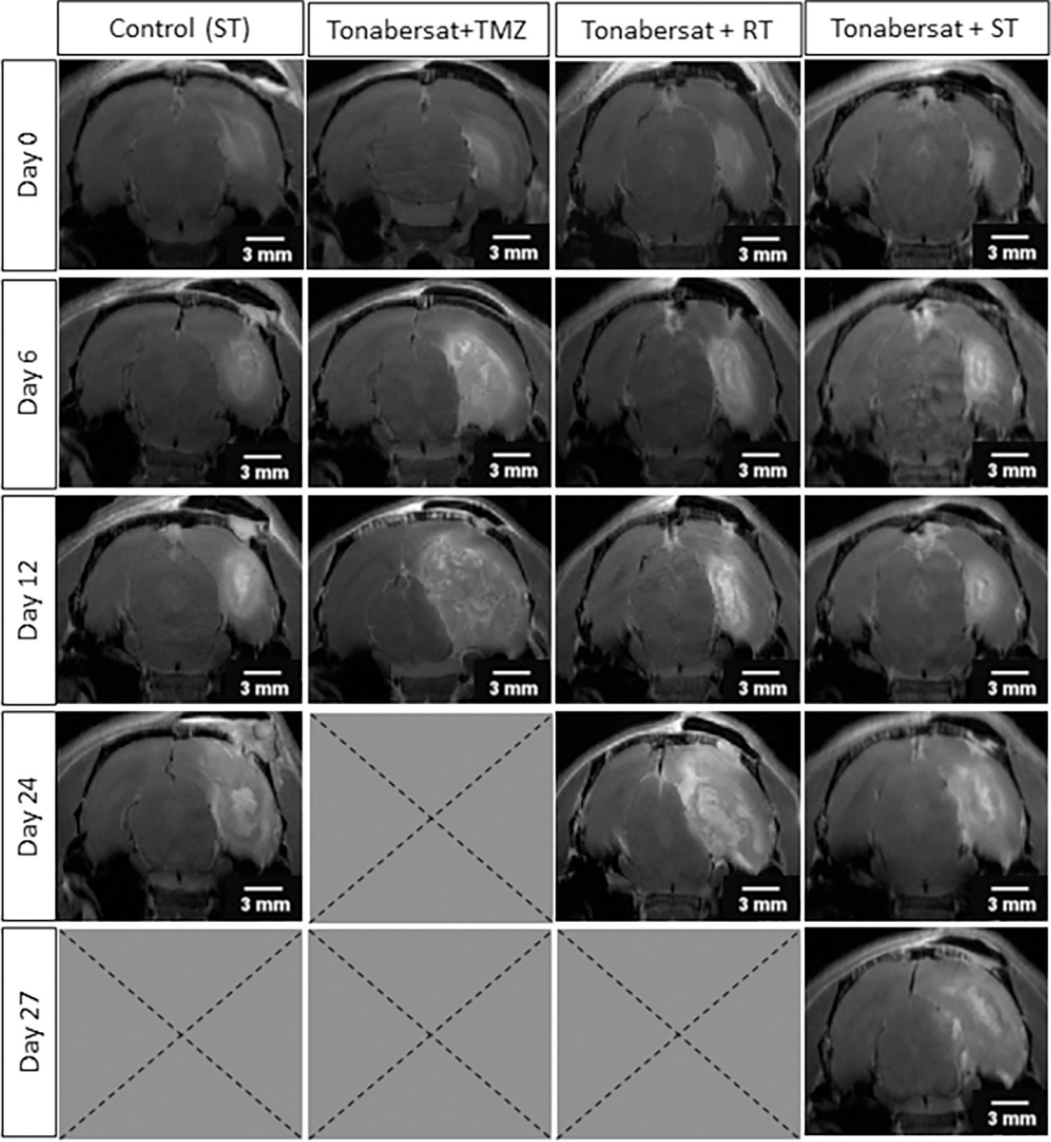
Visualization of the growth pattern of GB over time for the groups assessing the therapeutic potential of tonabersat. Key timepoints included: the day of confirmation (i.e., day 0), the day after fractionated radiotherapy is terminated (i.e., day 6), and when humane endpoints are reached (average euthanasia day varies per group). Abbreviations: standard of care (ST), temozolomide (TMZ), radiotherapy (RT). Scale bar: 3 mm.

Fig 2 illustrates the growth pattern of GB in one representative rat from each treatment group, utilizing CE T1-w MR images on key timepoints: the day of GB confirmation (i.e., day 0), the day after fractionated radiotherapy was terminated (i.e., day 6), and when humane endpoints were reached (average euthanasia day varies per group). Table 1 displays the estimated mean tumor volumes at these timepoints, complemented by their 95% confidence intervals.

**Table 1:** Estimated mean tumor volumes for the groups assessing tonabersat’s therapeutic potential. Key timepoints included: the day of confirmation (i.e., day 0), the day after fractionated radiotherapy is terminated (i.e., day 6), and when humane endpoints are reached (average euthanasia day varies per group). Abbreviations: temozolomide (TMZ), humane endpoints (H.E.).

Treatment group	Timepoint	Estimated GB volume	95% CI
**Control group**	Day 0	24.70 mm^3^	19.16–31.83 mm^3^
**Control group**	Day 6	39.75 mm^3^	32.33–48.86 mm^3^
**Control group**	Day 12	63.96 mm^3^	52.91–77.31 mm^3^
**Control group**	Day 24 (H.E.)	165.65 mm^3^	128.31–213.86 mm^3^
**Tonabersat + TMZ**	Day 0	24.22 mm^3^	17.12–34.27 mm^3^
**Tonabersat + TMZ**	Day 6	70.13 mm^3^	55.94–87.92 mm^3^
**Tonabersat + TMZ**	Day 12 (H.E.)	203.04 mm^3^	155.15–265.71 mm^3^
**Tonabersat + radiotherapy**	Day 0	29.01 mm^3^	22.79–36.92 mm^3^
**Tonabersat + radiotherapy**	Day 6	41.19 mm^3^	33.54–50.59 mm^3^
**Tonabersat + radiotherapy**	Day 12	58.50 mm^3^	48.47–70.60 mm^3^
**Tonabersat + radiotherapy**	Day 24 (H.E.)	117.98 mm^3^	95.21–146.19 mm^3^
**Tonabersat + standard of care**	Day 0	27.64 mm^3^	21.79–35.07 mm^3^
**Tonabersat + standard of care**	Day 6	40.85 mm^3^	33.28–50.15 mm^3^
**Tonabersat + standard of care**	Day 12	60.38 mm^3^	50.02–72.88 mm^3^
**Tonabersat + standard of care**	Day 24	131.91 mm^3^	107.19–162.33 mm^3^
**Tonabersat + standard of care**	Day 27 (H.E.)	160.37 mm^3^	128.33–200.41 mm^3^

### Evaluation of survival outcomes

**Fig 3:**
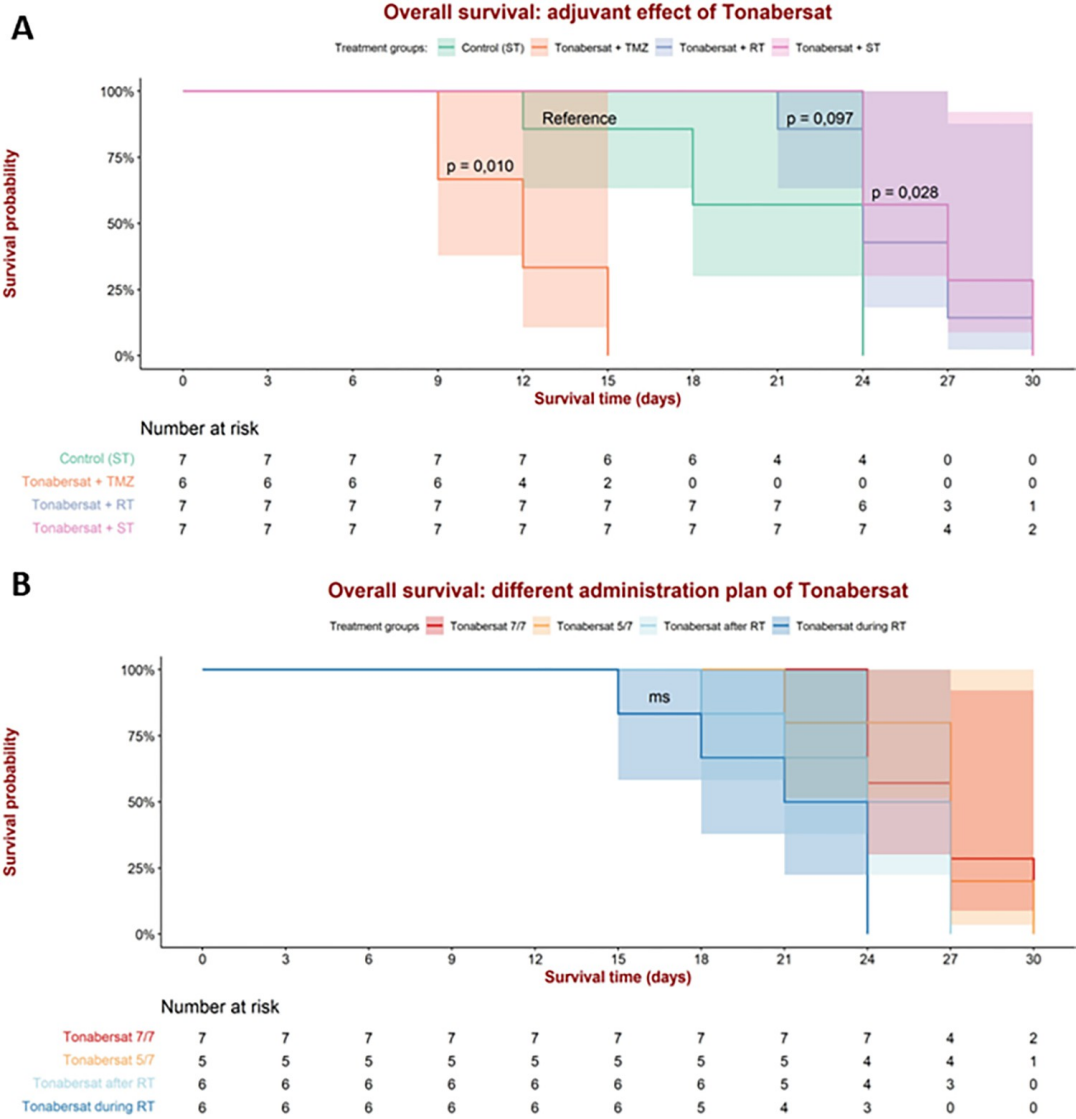
Kaplan-Meier survival curves for each treatment group alongside the respective number-at-risk table. Survival time is the interval between GB confirmation (i.e. day 0) and the humane endpoints (i.e. euthanasia, considered as the event). All animals reached the event. (A) The survival curves for the groups evaluating the adjuvant effect of tonabersat. Significant results were observed between the ‘control’ group (receiving standard care only) and the ‘tonabersat + TMZ’ group (p = 0.010) and ‘tonabersat + standard of care’ group (p-value = 0.028). Borderline significant results were observed between the ‘control’ group and the ‘tonabersat + radiotherapy’ group (p-value = 0.097). (B) The survival curves for the groups evaluating the various administration schedules for tonabersat. Marginally significant results were observed between the ‘tonabersat during radiotherapy’ group and the ‘tonabersat 7/7’ group (p-value = 0.054) and ‘tonabersat 5/7’ group (p-value = 0.098). Abbreviations: Standard of care (ST), radiotherapy (RT).

Fig 3 presents survival probability curves for each treatment group alongside the respective number-at-risk table. Survival time, defined as the time interval between GB confirmation (i.e., day 0) and the humane endpoints (i.e., euthanasia), is modeled as the time to event, where in this context the event is considered the euthanasia of an animal upon reaching humane endpoints. Notably, all animals in this study ultimately reached this event. The median overall survival for the different groups correspond to 27 days for the ‘tonabersat + standard of care’ group (i.e., ‘tonabersat 7/7’) and ‘tonabersat 5/7’ group, 24 days for the ‘control’ group, the ‘tonabersat + radiotherapy’ group, and the ‘tonabersat after radiotherapy’ group, 21 days for the ‘tonabersat during radiotherapy’ group, and 12 days for the ‘tonabersat + TMZ’ group.

In Fig 3A, the Kaplan-Meijer survival analysis reveals significant differences in survival probability between the ‘control’ group (i.e., receiving standard care only) and both the ‘tonabersat + standard of care’ group (p-value = 0.028) and ‘tonabersat + TMZ’ group (p-value = 0.010). The ‘tonabersat + TMZ’ group exhibited the lowest survival probability, with no animals surviving beyond 15 days post-tumor confirmation. 30% of animals in this group reached humane endpoints as early as day 9, making it the earliest event observed among the treatment groups, with half of the animals reaching the humane endpoints by day 12. In contrast, approximately half of the animals in the ’control’ group survived up to 18 days post-tumor confirmation. By day 24, all animals in the ’control’ group had been euthanized. In comparison, the ’tonabersat + standard of care’ group witnessed its earliest occurrence of euthanasia on day 24 post-confirmation, with approximately half of the animals reaching humane endpoints. This observation underscores the prolonged survival time when compared to the ’control’ group. Both the ‘tonabersat + standard of care’ and the ‘tonabersat + radiotherapy’ groups exhibited the longest survival probabilities, with 30% and 20% of animals, respectively, surviving until 30 days post-GB confirmation. A borderline significant result was observed between the ‘control’ group and the ‘tonabersat + radiotherapy’ group (p-value = 0.097).

In Fig 3B, no significant differences in survival outcomes were noted between the groups receiving tonabersat at varying administration schedules. Nevertheless, borderline significant results were observed between the ‘tonabersat during radiotherapy’ group and both the ‘tonabersat 7/7’ group (p-value = 0.054) and ‘tonabersat 5/7’ group (p-values = 0.098). Notably, the ‘tonabersat 7/7’ and ‘tonabersat 5/7’ groups had the longest survival duration, with animals surviving until day 30 post-tumor confirmation. In contrast, the ‘tonabersat during radiotherapy’ group and the ‘tonabersat after radiotherapy’ group had no survivors beyond day 24 and day 27, respectively.

### Therapy-induced changes in body weight and animal behavior

#### Body weight variation among treatment groups

**Fig 4:**
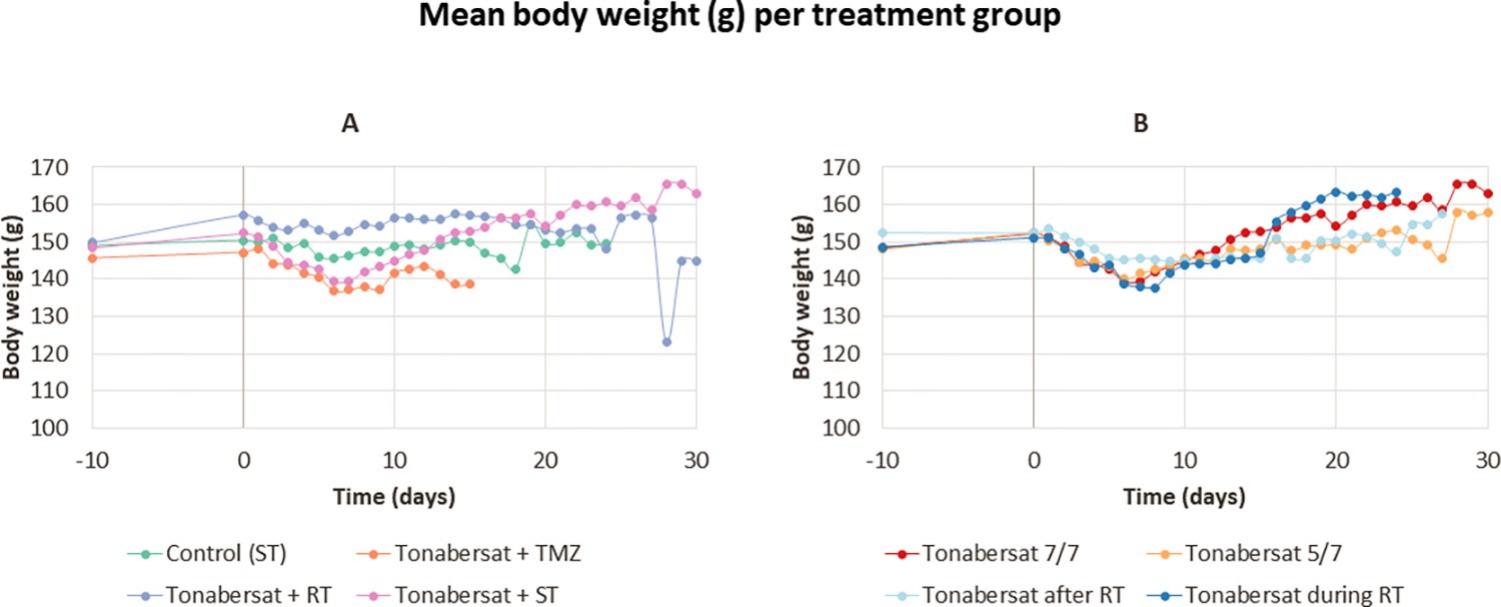
Mean body weight dynamics across treatment groups are illustrated over the course of the entire experimental period. Day -10 marks the day of inoculation, and day 0 corresponds to the day of confirmation. (A) Mean body weight alterations per treatment group, evaluating the tonabersat’s adjuvant potential. (B) Mean body weight changes per treatment group investigating the different administration schedules of tonabersat. Notably, animals receiving both tonabersat and TMZ injections undergo a significant weight reduction from day 1 to day 8. Following this reduction, the body weight of the animals in the ‘tonabersat + standard of care’ group stabilizes and subsequently surpasses its initial value. Conversely, groups receiving single injections (i.e., ‘control’ and ‘tonabersat + radiotherapy’ groups) exhibit consistent body weights throughout the entire experimental duration. Abbreviations: standard of care (ST), radiotherapy (RT), temozolomide (TMZ).

Fig 4 shows the mean alterations in body weight per treatment group evaluating tonabersat’s adjuvant potential (Fig 4A) and the different administration schedules of tonabersat (Fig 4B). In both graphs, day -10 marks the day of inoculation, and day 0 signifies the day of GB confirmation. Administration of both agents (i.e., tonabersat and TMZ) resulted in a significant reduction in body weight from day 1–5, which stabilized on day 8. Significant differences in body weight were observed between the ’control’ group and the ’tonabersat + TMZ’ group (p-value < 0.001), ’tonabersat + standard of care’ group (p < 0.0001), and the ’tonabersat + radiotherapy’ group (p-value < 0.0001). No significant differences were observed between the ’tonabersat + TMZ’ group and the ’tonabersat + standard of care’ group. Following stabilization in the ’tonabersat + standard of care’ group, body weight exceeded its maximum. Groups receiving a single agent (i.e., ’control’ group and ’tonabersat + radiotherapy’ group) did not display a significant reduction in body weight during days 1–8. Fig 4B confirmed these observations in the treatment groups that received different administration schedules of tonabersat. Detailed records of individual body weights for each animal within each treatment group are available in the supplementary data (S3 and S4 Figs).

#### Changes in animal behavior following standard therapy

**Fig 5:**
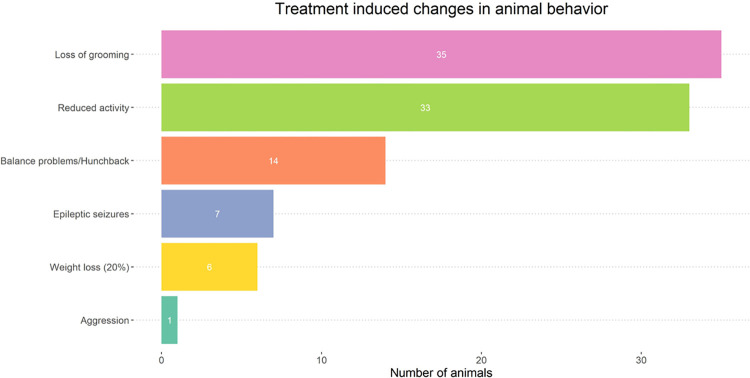
Visualization of behavioral changes in the F98 rat model post-GB development and standard therapy. Observed symptoms included grooming decline, reduced activity, balance problems, hunched-back posture, occasional epileptic seizures, and weight loss. A single instance of aggressive behavior was observed.

Fig 5 illustrates the manifestation of standard GB treatment on animal behavior. The most commonly observed symptoms across all groups receiving standard GB therapy included reduced activity levels and a decline in grooming behavior. Subsequent symptoms included balance problems, a hunched-back posture, weight loss, and occasional occurrences of epileptic seizures. A single instance of aggressive behavior was observed throughout the study.

### Histological evaluation

**Fig 6:**
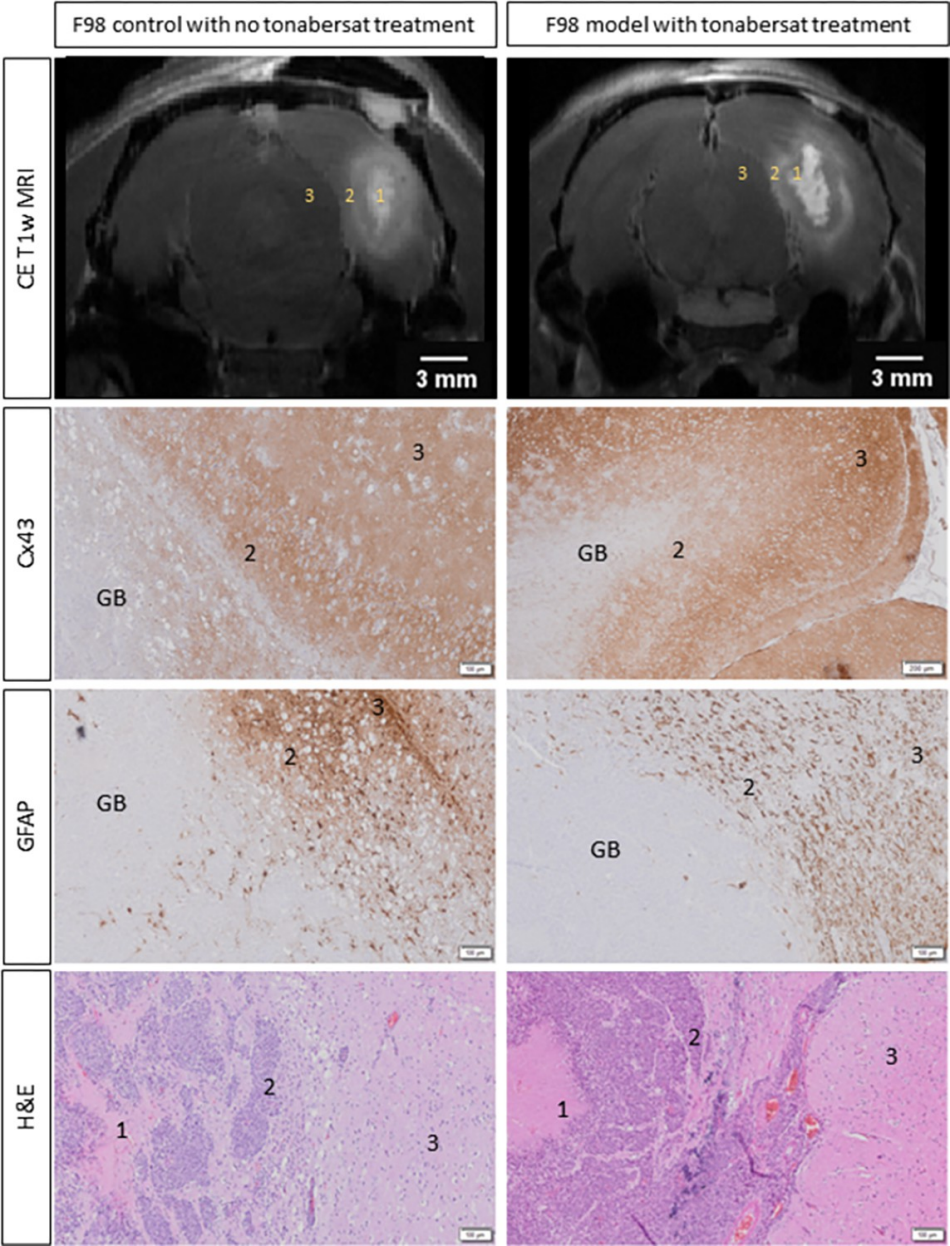
Visualization of the histological findings in F98 GB rats, accompanied with the corresponding CE T1-w MR images, comparing a control group without tonabersat treatment to a group receiving daily tonabersat injections. The histopathological examination, including H&E staining, revealed characteristic GB features with a necrotic tumor core (1) and a peritumoral zone containing infiltrating cancer cells (2) in the healthy brain tissue (3). Expression of Cx43 and the presence of GFAP-positive reactive astrocytes were observed in the peritumoral zone with infiltrating cancer cells but were absent in the necrotic tumor core.

Fig 6 presents histological data including H&E staining, Cx43 and GFAP immunostainings, and their corresponding CE T1-w MR images in a representative rat from the ‘control’ group (i.e., receiving standard of care, but no tonabersat treatment) and a rat from the ‘tonabersat + standard of care’ group (daily tonabersat treatment). H&E staining of paraffin-embedded brain sections from animals of each treatment group confirmed the presence of GB, characterized by a necrotic tumor core surrounded by a peritumoral zone containing infiltrating cancer cells within the adjacent healthy brain tissue. Expression of Cx43 and the presence of GFAP-positive reactive astrocytes were observed in the peritumoral zone with infiltrating cancer cells, but were absent in the necrotic tumor core. This observation highlights a specific association between Cx43 expression and GFAP-positive reactive astrocytes.

## Discussion

GB treatment typically involves a multimodal approach, outlined by Stupp et al. in 2005, including fractionated radiotherapy and TMZ chemotherapy, now recognized as the standard care regimen [9]. However, patient outcomes remain poor, necessitating the exploration of novel therapeutic approaches. Increasing evidence highlights the potential of combining therapies with the standard care due to the common occurrence of drug resistance and subsequent tumor recurrence with single-agent targeted treatments [37, 38]. With the aim of enhancing overall survival and prognosis for GB patients, this preclinical study investigates the potential of tonabersat as an adjuvant therapy. Recent studies have highlighted the crucial role of tumor microtubes, primarily involving Cx43 proteins, in glioma. These tumor microtubes facilitate direct communication between glioma cells and reactive astrocytes, contributing to brain invasion, proliferation, and therapy resistance, suggesting that inhibiting this intercellular communication could be a promising strategy to reduce glioma aggressiveness and enhance therapy effectiveness [11, 14, 20, 22]. In order to closely mimic human GB growth, it is imperative to employ a standardized animal model. Therefore, in this study, we utilized the optimized F98 GB rat model, which integrates the standard of care, to evaluate tonabersat’s adjuvant potential in the treatment of GB [39]. To mimic the clinical situation and evaluate brain tumor characteristics, including tumor volume, vascularization, BBB permeability, and responses to therapy, we used CE T1-w MRI [7, 43]. Additionally, we performed histological grading for GB diagnosis and analysis of GB characteristics. The stained sections revealed hallmark GB characteristics, such as infiltrative margins and the presence of a central necrotic core, affirming the model’s fidelity in approximating the aggressive nature of human GB [6].

GB growth exhibited a linear progression until approximately day 15 followed by an exponential increase from day 18 onwards when fractionated radiotherapy is combined with TMZ chemotherapy, which is consistent with previous descriptions by Zoteva et al. [39]. Importantly, all animals started the treatment protocol with similar initial tumor volumes (i.e., tumor volumes at day 0 did not differ significantly), allowing for a more controlled and comparable experimental environment and ensuring that treatment effects can be accurately assessed. Our results indicate a promising potential for tonabersat supplementation within the standard GB treatment protocol. Rats that received daily tonabersat alongside the standard treatment showed an extension in overall survival compared to the group that received fractionated radiotherapy in combination with TMZ chemotherapy. This extension manifested as an average survival increase of three to six days in rats, and when extrapolated to humans based on Sengupta et al.’s findings, this improvement corresponds to an enhanced overall survival of up to 7 months [44]. The relative extrapolated ages are dependent upon the life stage of a rat, for instance, one day in the life of a rat during adulthood is equivalent to approximately 35 human days [44]. Notably, the addition of TMZ to radiotherapy in human GB treatment has previously demonstrated a significant increase in overall survival of 2 months (i.e., from 12 to 14 months), underscoring the importance of novel adjuvant therapies like tonabersat in further improving outcomes for GB patients [9]. Should tonabersat prove effective in human trials, it holds promising prospects for substantial improvements in overall survival outcomes. Silberstein et al. conducted a safety assessment of tonabersat within a phase II clinical trial involving migraine patients [45]. The study reported only mild adverse effects, such as dizziness, nausea, and abdominal pain. In addition, animal studies illustrated no significant cardiovascular or central nervous system effects of tonabersat, even at very high doses [24, 45]. Also in our study, combining tonabersat with the standard of care demonstrated a more limited change in animal behavior.

De Meulenaere et al. reported significant differences between the tumor volumes in animals receiving the standard medical treatment and animals receiving standard medical treatment supplemented with tonabersat from day 15 onwards [32]. In contrast, we did not observe significant differences in tumor volumes between these two groups. However, by day 24 the tumor volumes in the group receiving standard therapy were 25% larger than those in the group were tonabersat was added to the standard of care, although no statistical significance was observed (p-value = 0.29). A possible explanation may lie in the use of two different regimens, i.e., fractionated radiotherapy (three fractions of 9Gy) as opposed to a single high dose applied in the study of De Meulenaere et al. (one fractions of 20Gy). The biological equivalence of three fractions of 9 Gy to the standard 30 fractions of 2 Gy, commonly applied in human therapy [9], potentially underlies the extended stabilization of GB growth observed in our study. The delivery of fractionated radiotherapy in multiple small doses over an extended period maximizes tumor cell damage while minimizing adverse effects on healthy brain tissue [46]. We consider fractionated radiotherapy as a crucial element of our treatment strategy, as it contributes to an extended stabilization phase of GB tumors through radiotherapy-induced apoptosis of GB cells. This observation is reinforced by the findings from the ’tonabersat + TMZ’ group, which displayed the poorest survival outcomes and failed to attain a stabilized tumor state, instead manifesting exponential tumor growth from the outset.

Cx43 appears to exert multifaceted involvement at various stages of glioma progression, influencing cell growth regulation, the facilitation of cell migration, and resistance to therapy-induced apoptosis. The dual role of Cx43 in tumorigenesis, acting as both a tumor promoter and a tumor suppressor, is partially due to its heterogeneous expression within the glioma during the different malignant stages [14, 47–49]. Cx43 expressing cells potentially impact tumor cell migration (i.e., Cx43 as tumor promoter), while Cx43 non-expressing cells may stimulate cancer cell proliferation (i.e., Cx43 as tumor suppressor) [47]. Therefore, using tonabersat to inhibit Cx43 at the tumor border has the potential to decrease the invasion of GB cells into the healthy brain tissue. Our histological analysis further supports this idea by confirming the presence of GFAP-positive astrocytes and Cx43 at the tumor border. Therefore, the use of tonabersat to inhibit Cx43 at the tumor border could potentially reduce the invasion of GB cells into the healthy brain tissue. These findings are in concordance with the research of McCutheon et al. and Sin et al., who demonstrated that the deletion of Cx43 diminishes the invasive capacity of GB [18, 19]. Interestingly, even with the incorporation of tonabersat into the standard of care (i.e., fractionated radiotherapy combined with TMZ chemotherapy), no significant differences in GB volumes were observed at a later stage when comparing the treatment groups to each other. This suggests that Cx43 inhibition may not impede tumor proliferation, however, tonabersat’s efficacy might lie in the peritumoral zone, influencing infiltrating cells and, consequently, playing a crucial role in inhibiting invasion into healthy tissue. Therefore, it is essential to explore alternative mechanisms beyond tumor volume measurements to elucidate the extended survival observed in our study. Despite the challenge in discerning changes in tumor volume, we did observe a significant difference in survival outcomes between the ‘control’ group and the ‘tonabersat + standard of care’ group, suggesting a beneficial impact of incorporating tonabersat into the treatment strategy.

TMZ is considered a radiosensitizer, increasing cellular sensitivity to radiotherapy and thereby enhancing its efficacy, our study indicates that tonabersat may exert a similar radiosensitizing effect [50, 51]. This effect is also evident in the ‘tonabersat + radiotherapy’ group, showing negligible differences in tumor volumes and survival outcomes compared to the ‘tonabersat + standard of care’ group. Also, it is worth mentioning that the addition of tonabersat to radiotherapy resulted in a prolonged survival time, with a trend toward significance (p-value = 0.097) compared to the use of TMZ in conjunction with radiotherapy (i.e., ‘control’ group). A hypothesis for tonabersat’s radiosensitization effect may involve the blockade of Cx43, potentially disrupting the therapy-resistant networks and leading to the accumulation of lethal levels of intracellular Ca^2+^ induced by radiotherapy, resulting in GB cell death [20, 21]. Furthermore, it has been observed that fractionated radiotherapy can enhance blood-brain barrier permeability to macromolecules in C6 glioma, potentially facilitating improved drug delivery to the brain, which could enhance the accessibility of tonabersat to the tumor site [52]. The study by Yusubalieva et al. also yielded prolonged survival in the C6 rat model when a monoclonal antibody targeting Cx43 was combined with radiotherapy. Conversely, when TMZ was combined with the same antibody, the anticancer effects were lost, leading to a shortened lifespan of the rats [53, 54].

To facilitate clinical translation, the administration schedule of tonabersat was also investigated in this study. Four treatment groups were used, all receiving standard treatment in addition to tonabersat, as this combination demonstrated optimal efficacy in the first part of the study. The ‘tonabersat 7/7’ group received daily tonabersat injections and was compared to a group receiving tonabersat five days a week (i.e., ‘tonabersat 5/7’). The selection of a two-day recovery period was grounded in the pharmacokinetics of tonabersat, which exhibits a median t_max_ absorption time ranging from 0.5 to 3 hours, and a terminal half-life of 24 to 40 hours [25, 55]. Astrocytic gap junctions play a pivotal neuroprotective role during oxidative and metabolic stress. *In vitro* studies have revealed that inhibiting astrocytic gap junctions enhances neuronal susceptibility to glutamate cytotoxicity [56]. Therefore, a two-day recovery period allows for the effective clearance of tonabersat, mitigating potential side effects, as the preservation of normal gap junction function is crucial for maintaining brain physiology and limiting neurological disorders [56]. Moreover, treatment schedules limited to weekdays are a common practice in clinical settings. In the standard therapy of GB, TMZ is orally administered to patients for five days during each 28-day cycle [9]. Hence, the treatment regimen involving tonabersat administered five out of seven days appears to align more suitably with clinical implementation objectives. In clinical practice, the paramount goal is to optimize patient treatment and enhance quality of life. If adopting a five-out-of-seven-day tonabersat administration regimen proves to reduce side effects in humans, and thus improve overall quality of life, it can provide a valid justification for opting for this schedule. In order to understand the impact of tonabersat at different stages of standard treatment, two additional groups were included, i.e., one receiving tonabersat during the radiotherapy protocol and a second group receiving tonabersat after the radiotherapy protocol. The assessment of tumor growth among the four treatment groups revealed no statistically significant differences. However, survival analysis indicated a borderline significant decrease in overall survival for the group receiving ‘tonabersat during radiotherapy’ when compared to both the ‘tonabersat 7/7’ group and the ‘tonabersat 5/7’ group. No significant differences in the overall survival between the ‘tonabersat during radiotherapy’ group, and the ‘tonabersat after radiotherapy’ group were observed, yet both groups displayed the lowest survival time when compared to the ‘tonabersat 7/7’ and ‘tonabersat 5/7’ group. In a randomized clinical trial by Spiro et al [57], assessing the impact of thoracic radiotherapy timing in patients with limited disease small-cell lung cancer, patients were randomly assigned to receive either early or late thoracic radiotherapy. This trial design is analogous to our ‘tonabersat during radiotherapy’ and ‘tonabersat after radiotherapy’ groups, which assess the early or late effects of tonabersat on standard treatment, especially in comparison to the continuous administration of tonabersat in the ‘tonabersat 7/7’ group. The main objective of the study by Spiro et al [57] was to underscore the significance of optimal chemotherapy delivery within the context of combined chemotherapy and irradiation. Despite not revealing a significant survival difference between the early and late thoracic radiotherapy groups, the trial demonstrated superior progression-free survival and overall survival in the early thoracic radiotherapy arm compared to a second similar trial. The main difference between the two trials seems to be associated with the ability to deliver chemotherapy as intended to the early thoracic radiotherapy arm. Their analysis suggests a potentially greater impact when all intended chemotherapy cycles are delivered, as some patients experienced nonhematologic toxicity or were too unwell to continue, leading to incomplete cycle delivery [57]. This is comparable to our findings, where continuous tonabersat administration (i.e., completion of all cycles) resulted in the most favorable survival outcomes. Interestingly, despite the radiosensitizing properties of tonabersat, discontinuing tonabersat administration after radiotherapy resulted in decreased overall survival compared to the ‘tonabersat 7/7’ group, highlighting the crucial role of continued tonabersat administration in extending survival. We postulate that the significance of continuous tonabersat administration may be linked to tonabersat’s short half-live and/or anti-epileptic mechanism of action [30, 31, 58]. The site of inoculation in our study, the right entorhinal cortex, is known for its propensity to induce epileptic seizures [59]. A substantial proportion of GB patients (30–62%) present with epilepsy, with an additional 20% developing it during later stages of the disease [58, 60]. Moreover, approximately 15–30% of GB patients experience drug-resistant epilepsy, which significantly impacts their quality of life [58, 61]. These epilepsy cases manifest in different forms; including focal or generalized tonic-clonic seizures [60]. In preclinical studies, tonabersat has demonstrated anti-seizure properties by significantly increasing the threshold for generalized tonic-clonic seizures [30]. After an initial period of seizure control with anti-epileptic drugs, seizures tend to reoccur or worsen due to GB progression, necessitating additional symptom management with anti-epileptic drugs [58, 61]. Our findings reveal some occurrences of epileptic seizures in the F98 GB model, visually monitored daily at consistent timepoints. Given tonabersat’s potential anti-seizure effects, continuous administration is recommended to mitigate epilepsy-related symptoms [30]. Noteworthy, a limitation in this study was the absence of personalized video-EEG monitoring for detecting epileptic seizures, which makes drawing definitive conclusions challenging. This proposition offers a promising avenue for future investigations, broadening its potential scope.

In summary, our study underscores the potential of tonabersat as a potent adjuvant to the standard of care for the treatment of GB in the F98 rat model, resulting in enhanced survival outcomes. Moreover, we propose that tonabersat demonstrates radiosensitivity, as indicated by the necessity of fractionated radiotherapy in the treatment regimen involving tonabersat. While the addition of tonabersat did not demonstrate a significant impact on tumor volumes (i.e., tumor proliferation) over time, it holds promise in mitigating tumor invasiveness. Minimizing tumor invasiveness has the potential to reduce post-tumor resection recurrence through more thorough GB debulking, ultimately leading to improved prognoses. Additionally, our findings recommend a five-out-of-seven-day dosing schedule for tonabersat as the optimal regimen for clinical translation, considering the potential benefits of a two-day recovery period on brain physiology. Continuous administration of tonabersat, both preceding and following radiotherapy, emerges as the most favorable approach for maximizing survival outcomes.

In conclusion, our preclinical investigation of tonabersat, a therapeutic compound administered orally in clinical trials involving over 1000 human subjects, highlights a potential advantage over other compounds. The demonstrated safety profile in humans makes tonabersat a promising candidate for further investigation as a potential adjuvant treatment for GB.

## Supporting information

S1 Fig Exponential growth of GB for each animal in the groups evaluating tonabersat’s adjuvant potential. (ZIP)

S2 Fig Exponential growth of GB tumor for each animal in the groups evaluating tonabersat’s administration schedule. (ZIP)

S3 Fig Body weight over time for each animal in the groups evaluating tonabersat’s adjuvant potential. (ZIP)

S4 Fig Body weight over time for each animal in the groups evaluating tonabersat’s administration schedule. (ZIP)

S1 Table Overview of humane endpoints for each animal. Rats were immediately euthanized when clinical and behavioral signs were observed.
(ZIP)
