# Supplementary figures and images for: Integrating and optimizing tonabersat in standard glioblastoma therapy: A preclinical study
Source: PLoS One. 2024 Mar 15;19(3):e0300552. doi: 10.1371/journal.pone.0300552 (PMC10942024; doi:10.1371/journal.pone.0300552)

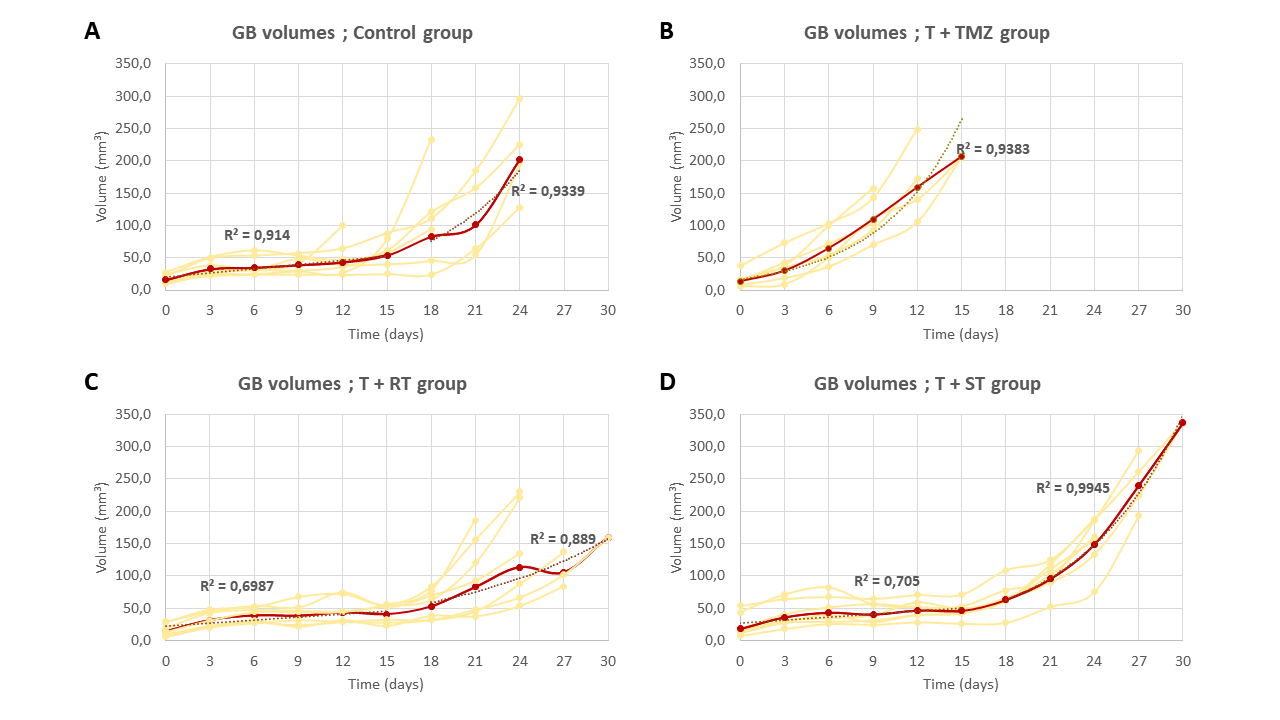

Supplement: S1 Fig — (ZIP) [file pone.0300552.s001.zip › S1_Fig1.tif]

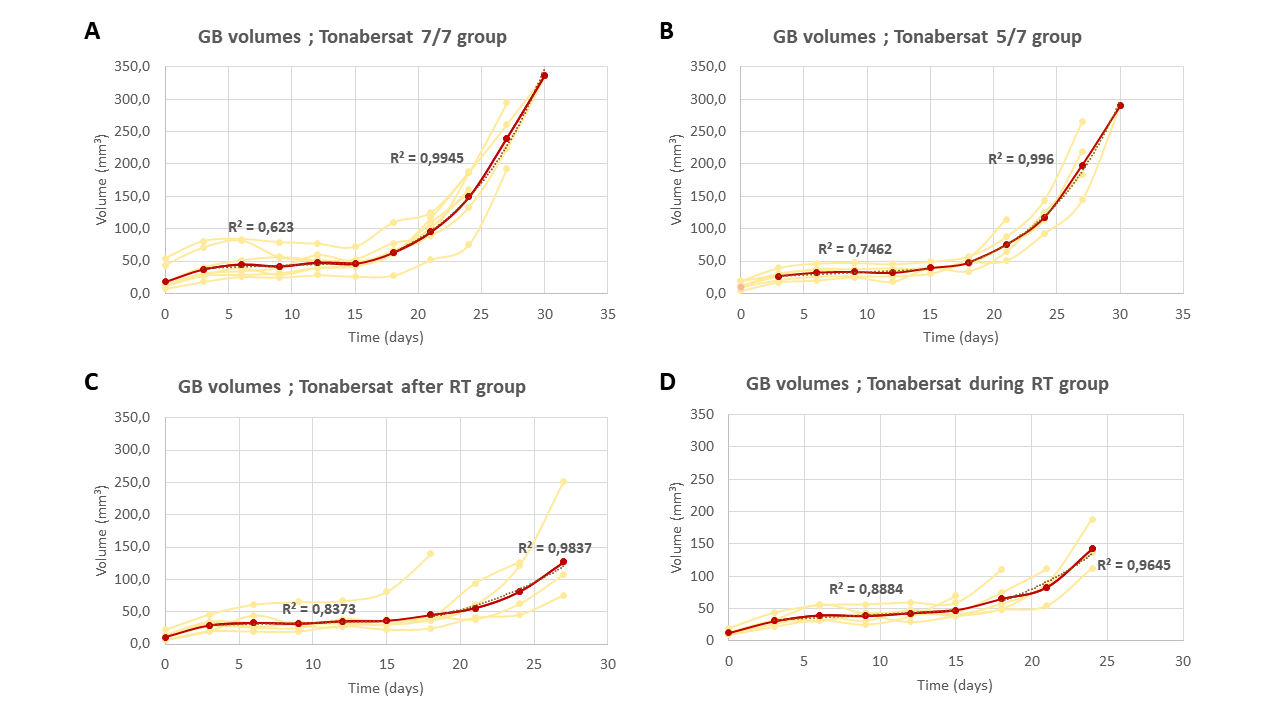

Supplement: S2 Fig — (ZIP) [file pone.0300552.s002.zip › S1_Fig2.tif]

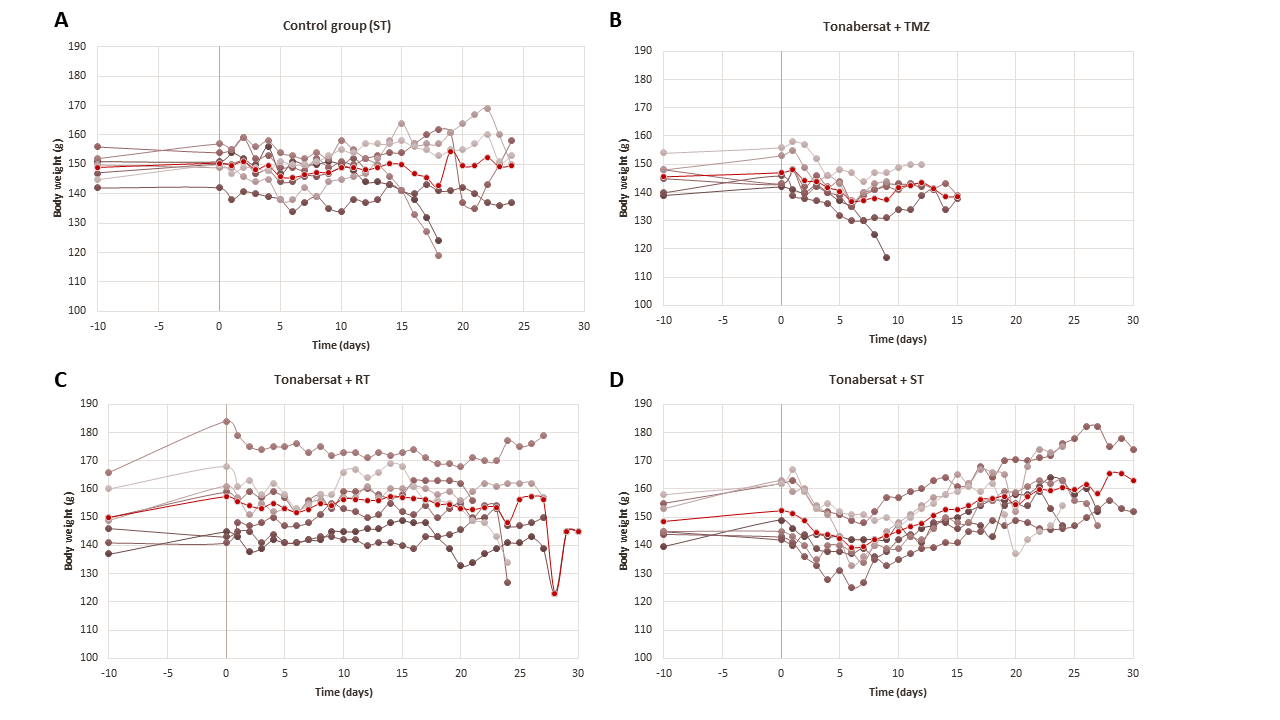

Supplement: S3 Fig — (ZIP) [file pone.0300552.s003.zip › S1_Fig3.tif]

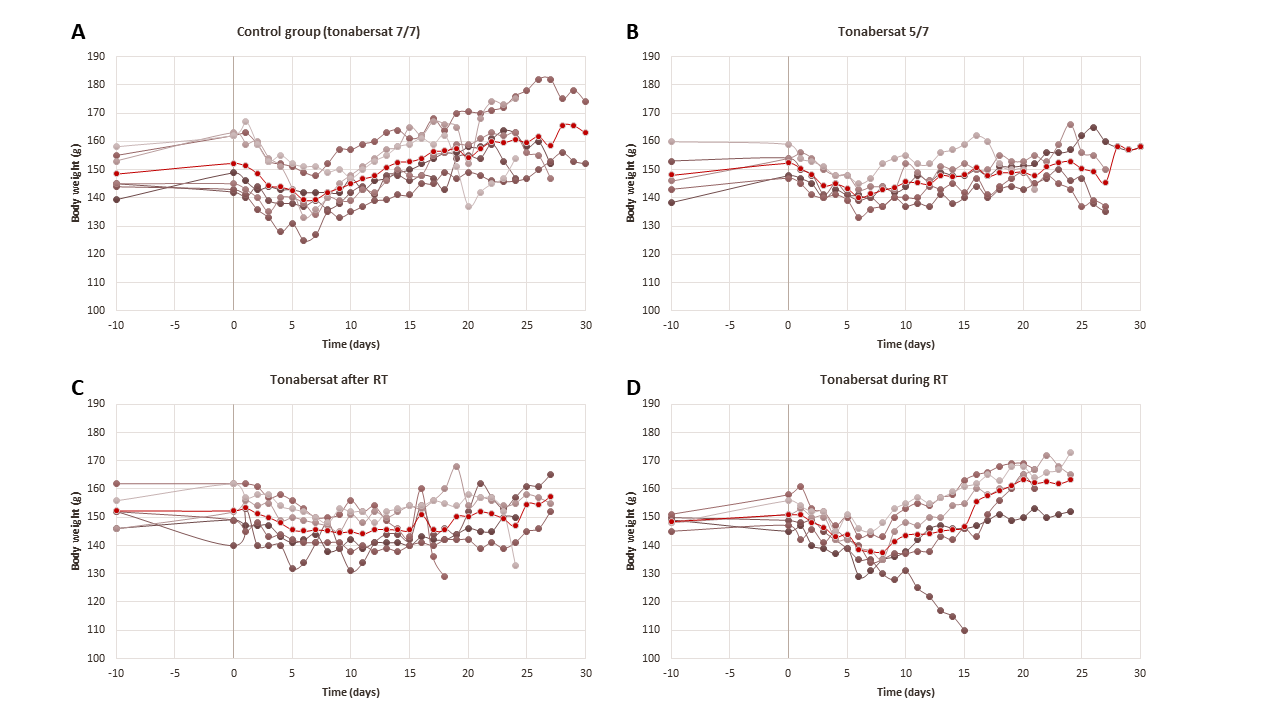

Supplement: S4 Fig — (ZIP) [file pone.0300552.s004.zip › S1_Fig4.tif]
